# 经支气管超声引导针吸活检在肺癌纵隔淋巴结分期的作用

**DOI:** 10.3779/j.issn.1009-3419.2010.05.04

**Published:** 2010-05-20

**Authors:** Eric LIM, Pallav L SHAH, 学博 李

**Affiliations:** 1 Imperial College and Academic Division of Thoracic Surgery, the Royal Brompton Hospital, London, United Kingdom; 2 Department of Respiratory Medicine, the Royal Brompton Hospital, London, United Kingdom; 3 山东中医药大学; 4 天津医科大学总医院，天津市肺癌研究所，天津市肺癌转移与肿瘤微环境重点实验室

纵隔淋巴结精确分期对于早期肺癌患者处理和手术路径的选择具有重要意义。

目前对于N2期肺癌的外科处理具有争议。临床医师目前比较多的看法是N2期肺癌由于手术治疗总体生存率低，所以不太适合手术。然而，随机试验结果显示，经诱导治疗后，手术与放疗后的总生存率无显著差异^[1-4]^。这些结果表明N2期肺癌患者的处理需要权衡各种疗法的利弊，同时也说明外科治疗仍是适合的选择方案之一。实际上，在Albain的研究中，诱导化-放疗后，外科手术组患者具有更好的无进展生存期^[4]^。

与其治疗选择一样，N2期肺癌的种类多种多样，从镜下肺癌到固定大块（Bulky）肺癌皆在此类。前者在开胸术前常难以诊断，后者则较少应用手术治疗。因此，绝大多数临床决定是基于N2肺癌病变部位的组织学结果和CT、PET或PET/CT检查标准。

气管旁组织包块的针吸活检诊断早在1978年便已开展^[5, 6]^，随着技术进步，更小的、具有更好弯曲性的穿刺针被制作用以进行弯曲支气管镜的穿刺抽吸^[5]^。随着支气管内超声（endobronchial ultrasound, EBUS）的发展，使得易于对纵隔及肺门淋巴结应用凸面探针进行EBUS实时引导下经支气管针吸活检（transbronchial needle aspiration, TBNA）。现在，EBUSTBNA已经越来越多地应用于肺癌或疑似肺癌患者的纵隔淋巴结分期。

我们最近于365本刊物中选择1950年-2008年刊发的符合入选标准的10项研究进行了系统评价和荟萃分析。EBUS-TBNA检测N2病变的点特异性为1.00（95%CI: 0.92-1.00），点敏感性为0.88（95%CI: 0.79-0.94）。EBUSTBNA的分辨力很好，通过受试者工作特征（summary receiver operating characteristics, SROC）曲线下区域为0.99（95%CI: 0.96-1.00）^[7]^。由于该技术包括组织活检，因此特异性可以达到100%，假阳性非常少见（即正常淋巴结误诊）。

虽然EBUS-TBNA具有很多优越性，但是其在肺癌分期的作用如何？发表的EBUS-TBNA对于CT或PET比较优势的研究结果，不一定意味着对已知或疑似肺癌患者进行纵隔淋巴结分期可以应用这项技术。目前仅有少数专家发表了相关研究证据，因此在大规模临床结果发表和临床专家认可之前很难得出结论。另外，EBUS-TBNA仅可以凭借气道临近的淋巴结状态，例如气管旁或隆突下淋巴结[虽然辅助应用内镜超声检查（endoscopic ultrasonography, EUS）可以探测更远区域]。

总之，一些临床医师应用PET/CT作为纵隔淋巴结分期的首选，采用TBNA “盲穿”、EBUS-TBNA和EUS技术确认或否定PET/CT发现。纵隔分期的精确性程度依赖于临床医师和患者对于N2疾病手术治疗的态度。如果对于PET/CT阳性N2疾病患者抱有可以手术态度的话，那么是否有必要进行采集更多组织标本的操作可能就有争议。另一方面，如果对手术治疗存在保守看法的话，那么主要精力应该投入确诊PET/CT发现的N2疾病。包括进行TBNA “盲穿”、EBUS-TBNA或EUS检查，以及可以进行针吸活检等具有100%特异性的操作，这样可以认为，只要患者结果阳性便意味着该处淋巴结有转移。但是，（多种技术）合并诊断敏感度为0.93，期望7%的N2病变患者得到假阴性结果。因此，如果EBUS-TBNA的结果为阴性，需要进行纵隔镜和淋巴结活检确证。在诊疗中期情况下，即手术在某些患者对最初诱导治疗反应情况基础上进行选择，这时，由于EBUS-TBNA操作难以获得足够的证据，其意义更难以评定。上述原因并不能改变EBUS-TBNA技术直观100%的特异性（确证N2疾病的能力），然而其敏感性（发现病变的能力）还没有定论。

EBUS-TBNA技术的引入为纵隔病变的分期提供了一个新的令人振奋的选择。其最佳作用依赖于适应征，专业程度以及最重要的是，对于N2疾病患者（外科）治疗的态度。
